# Shock wave lithotripsy as a primary modality for treating upper ureteric stones: A 10-year experience

**DOI:** 10.4103/0970-1591.44253

**Published:** 2008

**Authors:** Abhijit S. Padhye, Pushkaraj B. Yadav, Pratikshit M. Mahajan, Ashish A. Bhave, Yogesh B. Kshirsagar, Yogesh B. Sovani, Shivadeo S. Bapat

**Affiliations:** Dr. Y. G. Bodhe, Dept of Urology; Maharashtra Medical Foundation and Maharashtra Medical Research Centre, 986 Senapati Bapat Road, Pune - 411 053, India

**Keywords:** Shock wave lithotripsy, upper ureteric stone

## Abstract

**Aims and Objectives:**

Shock wave lithotripsy (SWL) has been recommended as a first-line treatment for upper ureteric calculi in several studies with a success rate of 80-90%. Our aim is to present our retrospective data of treatment of upper ureteric stones with SWL as primary modality over a 10-year period and evaluate the factors affecting fragmentation and clearance.

**Materials and Methods:**

From February 1997 to March 2007, 846 patients with upper ureteric stones were treated with SWL as the primary modality. Age: 9-69 years, 546 males and 300 females, stone size: 7-22 mm. Pyuria in 132/846 with clinical infection 40/132, pre-SWL JJ stenting: 40/846 and anesthesia in 41/846 patients. Duration of symptoms: < 4 weeks- 780/846, >4 weeks- 66/846. Stone size: < 1 cm- 513/846, >1 cm-333/846. Workup: X-Ray KUB, Urine and Uro-USG. Intravenous urogram (IVU): 130/846. Intraoperative (C-arm) fluoroscopic imaging was used. Presentation: colic-801/846, incidental-45/846. Criteria for clearance: symptomatic relief, X-ray and USG confirmation.

**Results:**

Clearance rate: < 1cm- 95.91% (492/513), >1 cm- 85.29% (284/333). Overall clearance rate: 91.73% (776/846). No clearance: 70/846 (8.27 %). In these, 59/70 underwent ureteroscopy, 8/70 percutaneous nephrolithotomy and 3/70 open ureterolithotomy for clearance. Post SWL complications were seen in 25 (3%) cases with septicemia in nine and stein strasse in 16 cases. Duration of symptoms < 4 weeks - 93.7% success (731/780), >4 weeks – 68.1% (45/66). Non-stented – 92% (744/806) success. Stented group–80% (32/40).

**Conclusions:**

Best results with SWL as monotherapy for upper ureteric stones are achieved when stones are less than 1 cm in size, of short duration history and without indwelling stents. Overall success rate – 91.73%.

## INTRODUCTION

The management of calculi in the urinary tract has been revolutionized by the introduction of extracorporeal shock wave lithotripsy (SWL) by Chaussey *et al.* in the early 1980s.[1] SWL has been recommended as a first-line treatment for upper ureteric calculi in several studies with a success rate of 80-90%.[2–4] Ureteroscopy and intracorporeal lithotripsy is used as a salvage procedure. The success of lithotripsy depends on stone composition, position and size.[5] With the advent of small-caliber and flexible ureteroscopes, the paradigm of treatment of upper ureteric stones has shifted towards ureteroscopy with success rates approaching 95% but not without its share of complications. SWL, on the other hand, is noninvasive and less morbid with a low complication rate. We would like to present our retrospective data of treatment of upper ureteric stones with SWL as primary modality and evaluate the factors affecting successful fragmentation and clearance.

## MATERIALS AND METHODS

From February 1997 to March 2007, 846 patients with upper ureteric stones were treated with SWL using Dornier Compact S lithotripter. Upper ureter is defined as part of the ureter between the pelvi-ureteric junction and the sacro iliac joint. Mean age was 41 years (range 9 to 69 years). Series included 546 males (66%) and 300 females (34%). Out of 846 patients, 801 presented with colic, while in 45 patients it was an incidental finding. Duration of symptoms was less than four weeks in 780/846 patients (92%) and more than four weeks in 66/846 (7.8%) patients. Prior to SWL all patients were investigated for urine routine and culture, serum creatinine and coagulation profile according to hospital protocols. Mean serum creatinine was 1 mg%(0.7-1.8). Pyuria was seen in 132/846 (15.6%) patients with positive cultures seen in 40/846 (4.7%) patients. All these patients were treated with antibiotics at least 48 h before the procedure. Radiological investigations in the form of X-ray KUB and uro-ultrasound were done in all cases to identify number, site and size of stones and presence of back pressure changes in the form of hydronephrosis. Intravenous urography was performed in 130/846 (15.3%) patients. Patients with small shrunken kidney with doubtful function, abnormal coagulation profile and chronic renal failure were not included in the study. Preoperative JJ stenting was done in 40/846 (4.7%) patients. Indications for stenting were calculus anuria (12) and severe degree of obstruction (28). All patients were treated on outpatient basis except for 12 cases that presented with calculus anuria; 10/12 patients had solitary functioning renal unit while two had bilateral ureteric (right upper and left lower ureteric) stones. All of these patients were stented before undergoing SWL. SWL was carried out when nadir creatinine value reduced < 2 cm with documented voided volume of > 2 liters. Mean stone size was 9.8 mm (7-22 mm). While 513/846 (60.6%) patients had less than 1 cm stones, stones more than 1 cm in size were seen in 333/846 (39%) patients. All patients underwent SWL in supine position. Stone localization was done using C-arm (fluoroscopy imaging). Anesthesia was required in 41 cases (five- pediatric cases, 36- intolerable pain during SWL). Mean number of shocks per stone were 2500 with mean intensity being 5. Patients were followed up with X-ray KUB at 15 days and if incomplete fragmentation was noticed repeat sitting of SWL was given. Patient was termed as SWL failure when incomplete or no fragmentation was found after three sittings. Criteria for clearance were symptomatic relief, absence of residual fragments on X-ray KUB at three months and reduction of stasis in the proximal tract as seen on follow-up USG.

## RESULTS

In our series overall stone-free rate at three months was 91% (776/846). Clearance after first sitting was 41% (347/846), after second sitting was 30.7% (260/846) and after third sitting was 19.9% (169/846). Clearance according to size: < 1 cm – 95.9% (492/513), > 1cm – 85.2% (284/333) [Table 1]. Eight per cent (70) cases did not have successful outcome. Of these, 50 cases had incomplete fragmentation and were termed as SWL failures. They required auxiliary procedures in the form of URS (39), PCNL (8) and open ureterolithotomy (3). Remaining 20/70 cases had effective fragmentation but incomplete clearance and underwent URS with stone extraction for the same. In the preoperatively stented group 8/40 (20%) required auxiliary procedures (URS) for clearance. Post SWL complications were seen in 25 (3%) cases with fever, septicemia in nine cases which was treated with culture-specific antibiotics and stein strasse with colic in 16 cases. All these patients had stones larger than 1 cm. Six cases required URS and extraction of lead fragment while 10 cases passed fragments on their own.

**Table 1:** Results

Clearance	1^st^ Sitting	2^nd^ Sitting	3^rd^ Sitting
	347/846 (41.01%)	260/846 30.7%)	169/846 (19.9%)

### Statistical analysis of results

Of the total success cases (776) a significant proportion of patients (63.4%) had stone size smaller than 1 cm while of the total failures (70) a significant proportion (70%) had stone size greater than 1 cm. [Table 1]. Mean stone size was significantly (*P*<0.00) smaller for group (1.37±0.42cm) when tested using t test. Patients with smaller stone size needed significantly (*P*<0.00) smaller number sittings compared to those with larger stone size when tested by t test [Table 2]. Stone size was significantly (*P*<0.00) larger among patients who had duration of symptoms more than four weeks (1.44±0.25) compared to those who had duration of symptoms less than four weeks (1.05±0.38) (using t test.). Clearance was dependent on stone size among patients with short as well as longer duration of symptoms. For 1 cm increase in stone size, number of sittings increased by 1.38 among patients with shorter duration of symptoms and by 1.82 for longer duration of symptoms [Table 3]. Logistic regression was done for predicting the risk of failure and showed that risk was significantly higher (Odds ratio 3.3, 95% CI 1.8 – 6.0, *P*< 0.00) if stone size was greater than 1 cm . Similarly, risk was significantly higher (Odds ratio 3.4, 95% CI: 1.8 – 6.5, *P*< 0.00) if duration of symptoms was higher than four weeks.

**Table 2:** Mean number of sittings by stone size

Stone size	Sittings	significance
< 1 cm	1.51 ± 0.65	*P* < 0.000
> 1 cm	2.21 ± 0.79	

**Table 3:** Regression analysis for clearance and stone size

Dependent variable	Duration (weeks)	n	Regression coefficient for stone size (β ± se)	R^2^ (%)
Clearance	<4 weeks	731	± 0.06	43.7
(sittings)	>4 weeks	45	1.82 ± 0.41	30.4

## DISCUSSION

Multiple treatment modalities are available for upper ureteric stones such as: 1. URS 2. PCNL 3. SWL 4. Open surgery. Amongst these SWL has very good success rates and high degree of patient satisfaction.

We had an overall stone-free rate of 91.7%. This result compares favorably with previously published series and is a timely reminder that good stone-free rates can be achieved without the use of ureteroscopy. Previous studies with different lithotriptors reported success rates between 80-90%.[6] In the study of Gnanapragasam *et al.*[4] , stone-free rates for upper ureteric stones were 90%. Failure of SWL was seen in patients with stone size >1.3 cm. Similarly, Mogensen and Anderson[3] reviewed outcomes of 199 patients with ureteric stones treated with SWL. Stone-free rates at three and six months after SWL for upper ureteric stones were 86% and 91% respectively. Hofbauer *et al.*[7] evaluated the outcome of 1259 ureteric stones with success rate of upper ureteric stones being 98%.

We had retreatment rate of 59% and auxiliary procedures were required in 8% cases. Fetner *et al.*[8] found a statistically significant relation between stone size and success rate. The American Ureteral Stones Clinical Guidelines Panel[9] reported that, for proximal ureteric stones, the success rate of SWL was 87% for <1 cm stone and 76% for >1 cm stone. In our study 95% success was seen in cases with <1 cm stone while 85% success was seen in >1 cm stone. This success rate may be due to better stone localization techniques and use of standard lithotripter (Dornier Compact S lithotripter). Duration of symptoms also affects the outcome of treatment. Longstanding stones had more retreatment and failure rates. These impacted stones have a lot of surrounding mucosal edema and hence these stones have incomplete clearance. This was confirmed during open ureterolithotomy procedures where it was found that the stone was completely fragmented with SWL but the fragments were not cleared due to edema of surrounding mucosa. Of 66 cases with duration of symptoms of > four weeks, 21 (31%) cases required auxiliary procedures.

Pushback technique was not used in any of our patients. All stones were treated without manipulating the position of the stone. There is no significant difference in success rates for in situ versus pushback SWL.[7,10,11] Macroscopic expansion space is not required for successful fragmentation of ureteric calculi.[12,13] Ureteral manipulations using pushback technique are associated with 5.1% perforation rate.[10]

We also observed that the presence of JJ stents significantly reduces the success rates. JJ stents were inserted in 40 cases preoperatively of which eight (20%) patients required auxiliary procedure in the form of ureteroscopy. Ryan *et al.*[14] showed that *in situ* ureteric stents impair ureteric peristalsis and trap large fragments thus delaying stone clearance. Presence of JJ stent next to the stone may prevent full impact of the shock wave on the stone. However, JJ stents are a must in stones with severe obstruction or solitary functioning renal unit.[15]

Anesthesia was required in 40 cases only, of which five were children below 12 years of age. Mean intensity of shocks was 5, which increased to 7 during anesthesia thus effecting fragmentation.

In 1997 the AUA published its recommendations that for stones greater than 1 cm in the proximal ureter SWL, PCNL and ureteroscopy were all acceptable approaches.[7] Currently there seems to be a shift away from noninvasive SWL in favor of more invasive ureteroscopy options.[16] This is because of significant advances made in ureteroscopic technology, with development of smaller caliber and flexible scopes. Also available are better stone-breaking systems (laser, efficient lithotripsy probes). Thus the success rates of ureteroscopy for upper ureteric stones approach 90–95%. But ureteroscopy is a more morbid procedure with increased hospitalization and higher complication rate. Even with small-caliber scopes ureteric perforation rates are 0-5% and stricture rates 1-4%.[17,19] Conversely SWL has almost similar success rates of 91% in our study with low complication rate and failure rate with far better patient acceptance.

## CONCLUSION

The results of our study show that SWL as a primary modality for upper ureteric stones has an overall success rate of 91%. Success rate drops with increasing size of stone, duration of stone in ureter and presence of indwelling JJ stents. With availability of newer machines minimal or no anesthesia is required.
